# Protocol for preparing formalin-fixed paraffin-embedded musculoskeletal tissue samples from mice for spatial transcriptomics

**DOI:** 10.1016/j.xpro.2024.102986

**Published:** 2024-03-29

**Authors:** Esther Wehrle, Denise Günther, Neashan Mathavan, Amit Singh, Ralph Müller

**Affiliations:** 1 Institute for Biomechanics, ETH Zurich, Zurich, Switzerland; 2 AO Research Institute Davos, Davos Platz, Switzerland

**Keywords:** Health Sciences, Genomics, Microscopy, Molecular Biology, Molecular/Chemical Probes

## Abstract

Here, we present a protocol for using spatial transcriptomics in bone and multi-tissue musculoskeletal formalin-fixed paraffin-embedded (FFPE) samples from mice. We describe steps for tissue harvesting, sample preparation, paraffin embedding, and FFPE sample selection. We detail procedures for sectioning and placement on spatial slides prior to imaging, decrosslinking, library preparation, and final analyses of the sequencing data. The complete protocol takes ca. 18 days for mouse femora with adjacent muscle; of this time, >50% is required for mineralized tissue decalcification.

For complete details on the use and execution of this protocol, please refer to Wehrle et al.^1^ and Mathavan et al.^2^

## Highlights

• Steps for spatial transcriptomics of FFPE musculoskeletal samples from mice
• Instructions on sample decalcification prior to spatial transcriptomics
• RNA quality required for spatial transcriptomics is maintained

## Abstract (editor highlights)

Publisher’s note: Undertaking any experimental protocol requires adherence to local institutional guidelines for laboratory safety and ethics.

## Graphical abstract

**Figure:**
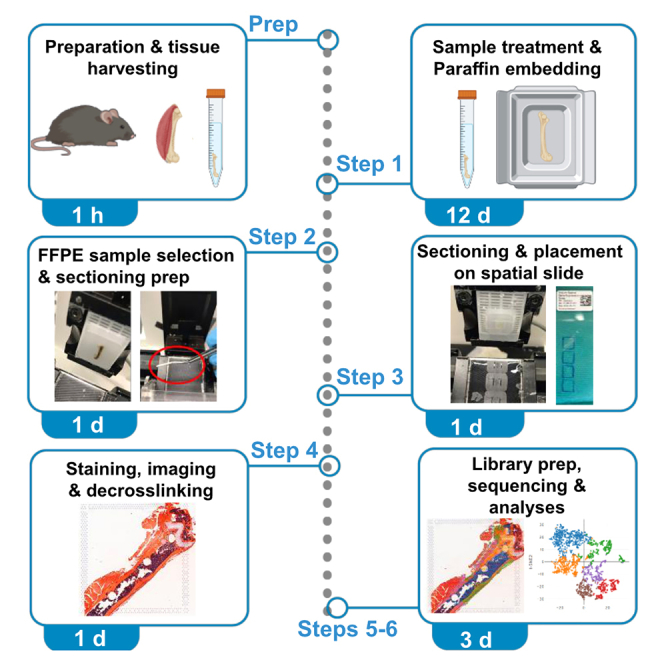

## Before you begin

Bone adaptation and healing are spatially and mechanically controlled processes^3^ involving crosstalk of multiple tissues.^4^ Preservation of the spatial context represents a transformative advancement in cellular profiling technologies (e.g., RNA sequencing) with the potential to supplant established histology-based methods, such as *in situ* hybridization, immunohistochemistry and dissociated single-cell techniques. Whereas spatial transcriptomics analyses have successfully been applied in many research areas,^5^ its use in bone has been limited due to the calcified nature of the samples often associated with tissue detachment from slides. Recently, spatial transcriptomics approaches have been described for cryo-embedded skeletal samples from P0 calvaria and during digit regeneration in mice.^4^^,^^6^^,^^7^ To allow for broader application of spatial transcriptomics in musculoskeletal research, we have now established a protocol for FFPE calcified tissue samples from mice.

Here we describe a detailed protocol that permits the use of spatial transcriptomics (Visium, 10× Genomics) in bone and multi-tissue musculoskeletal FFPE samples from mice. The protocol below describes the specific steps for samples from an externally-stabilized femur defect model in mice. However, we have also used this protocol for bone samples (vertebra, intact femur), muscle samples (m. quadriceps femoris) and multi-tissue samples (intact femur with adjacent muscle). The protocol is structured into seven steps ranging from tissue harvesting and preparation to the final analyses of the sequencing data. The complete protocol takes ca. 18 days for mouse femora with adjacent muscle; of this time >50% is required for mineralized tissue decalcification. Potential pause points are indicated in the detailed description of the procedure.

### Institutional permissions

The FFPE embedded musculoskeletal tissue samples used for protocol establishment were obtained from fracture healing experiments in mice. For these experiments the mice were either purchased from the distributor Janvier (Saint Berthevin Cedex, France) or bred in house at the ETH Phenomics Center (EPIC). All animal procedures were approved by the Commission on Animal Experimentation (license numbers: ZH181/2015 and ZH229/2019; Kantonales Veterinäramt Zürich, Zurich, Switzerland). We confirm that all methods were carried out in accordance with relevant guidelines and regulations (Swiss Animal Welfare Act and Ordinance (TSchG, TSchV)) and reported considering ARRIVE guidelines. Animal experiments were performed using previously established protocols for osteotomy surgery, analgesia/anesthesia, post-operative monitoring by *in vivo* time-lapsed micro-CT imaging and euthanasia of the animals for tissue sampling.^8^^,^^9^^,^^10^

Prior to starting this protocol, all relevant permissions for performing animal experiments and for obtaining musculoskeletal samples from mice need to be acquired.

### Preparation and tissue harvesting

**Timing: 1 h**

1. Preparation.
  a. Wear gloves.
  b. Fill Falcon tubes with ca. 12 mL of 10% neutral buffered Formalin solution and place them on ice add Formalin concentration.
  c. Wipe all table surfaces with 70% Ethanol (table 1 for animal euthanasia; table 2 for sample prep).
  d. Place and fix anesthesia mask on table 1.
  e. Place heating mat on table 1.
  f. Cover heating mat with drape or paper towel.
  g. Place shaver and large scissors/tweezers, +/- lancets on table 1.
  h. Place a sterile Foliodrape on table 2 and tape at edges to the table.
  i. Fill plastic box with ice, 1 cm below edge, flatten ice down to have a flat surface.
  j. Place box filled with ice in middle on Foliodrape without touching other areas of drape.
  k. Place 1 Petri dish (closed) on ice.
  l. Place autoclaved surgical instruments (scissors, tweezers, scalpel blade) next to box on Foliodrape in a sterile manner.
  m. Prefill anesthesia induction box with isoflurane (3%).
2. Tissue harvesting.
  a. Place mouse in induction box until asleep with slow respiration pattern.
    ***Optional:*** Put on two pairs of sterile gloves.
    ***Optional:*** Take the body weight of the animal.
    ***Note:*** Unexpected body weight changes may give indications to exclude an animal from Spatial Transcriptomics experiments.
  b. Place mouse on table under inhalation anesthesia via mask.
    ***Optional:*** Take blood via heart puncture or facial vein (freckle).
    ***Note:*** Blood analyses can be used as an inclusion/exclusion criteria for an animal to Spatial Transcriptomics.
  c. Turn the isoflurane concentration to maximum.
  d. Shave the right and left femur + the distal part of the back of the mouse.
  e. Euthanize the animal, e.g., by cervical dislocation or decapitation.
  f. Visually confirm complete disconnection of tissue.
  g. Place mouse on Petri-dish on ice (second table).
  h. Make circumferential skin incision with scissors around right femur.
  i. Incise muscles of upper hindlimb toward hip joint.
  j. Ex-articulate hip joint via manually rotating.
  k. Remove right hind limb and place on lid of Petri dish.
  l. Remove outer pair of gloves.
  m. Hold the tibia with tweezers on the Petri dish (cranial part of tibia facing down).
  n. Use a scalpel (scalpel blade 11 or 15) to disconnect the tibia and the femur in the knee joint.
  o. Place femur +/- surrounding muscle in pre-cooled formalin solution.
    ***Note:*** Remove as much muscle tissue surrounding the femur as wanted.
    ***Note:*** Femur and m. Quadriceps can be kept together throughout decalcification.
    ***Note:*** if using bones from fracture healing studies with external fixation, the fixator can either be removed now or kept in place until Paraffin embedding.
    **CRITICAL:** Steps h-o should be done as fast as possible (max. 10 min) to avoid RNA degradation.

## Key resources table

**Table:** 

REAGENT or RESOURCE	SOURCE	IDENTIFIER
**Biological samples**		

Musculoskeletal tissue samples from mice	This paper, ETH Phenomics Center (EPIC)	N/A

**Chemicals, peptides, and recombinant proteins**		

Formalin solution, neutral buffered 10%	Sigma-Aldrich	HT501128
EDTA	Sigma-Aldrich	ED-1KG
PBS	Medicago	09-9400-100
Paraffin for tissue embedding	Leica Biosystems	39602012 Surgipath Paraplast
Ethanol, molecular grade	Fisher BioReagents	BP2818-100
RNase away	n.p.	n.p.
Milli-Q water	Millipore	n.p.
All additional material as listed in user guide	10× Genomics	CG000407 | Rev D

**Critical commercial assays**		

RNeasy FFPE kit	QIAGEN	73504
Visium Spatial Gene Expression Slide Kit	10× Genomics	PN-1000188
Visium Mouse Transcriptome Probe Kit	10× Genomics	PN-1000365

**Experimental models: Organisms/strains**		

C57BL/6J mice, 16–27 weeks, female	Janvier	N/A
BCR mice (Ibsp/Acp5): C57BL/6j.Ibspem1(ETHZ).Acp5em1(ETHZ), 16 weeks, female	ETH Phenomics Center (EPIC), ETH Zurich	N/A

**Software and algorithms**		

Space Ranger (version 2.0.0)	10× Genomics	https://support.10xgenomics.com/spatial-gene-expression/software/downloads/latest
Mouse reference genome	Ensembl 98, version M23	https://cf.10xgenomics.com/supp/spatial-exp/refdata-gex-mm10-2020-A.tar.gz
Slide layout general purpose raw (GPR) file format	10× Genomics	https://support.10xgenomics.com/spatial-gene-expression/software/pipelines/latest/using/slidefile-download
Probe set reference CSV file	10× Genomics	https://cf.10xgenomics.com/supp/spatialexp/probeset/Visium_Mouse_Transcriptome_Probe_Set_v1.0_mm10-2020-A.csv
H&E stained image of capture area	This paper	N/A
Seurat package (version 4.2.0)		https://satijalab.org/seurat/
Loupe Browser (version 6.3.0)	10× Genomics	https://www.10xgenomics.com/support/software/loupe-browser/latest
ZEN 2.6 (blue edition)	Zeiss	https://www.micro-shop.zeiss.com/en/us/softwarefinder/software-categories/zen-blue/

**Other**		

Foliodrape	Hartmann	277 5032
Swabs, sterile	Covetrus	2800224
Marker, water + Ethanol resistant	VWR	52877-310
Scalpel blades, 15, 11	n.p.	n.p.
Autoclave bags	n.p.	n.p.
Lancets for bleeding	n.p.	n.p.
Sterile gloves	n.p.	n.p.
Isoflurane	n.p.	n.p.
Histology cassettes	Simport	M515-2
Square box wrench	RISystem	R1S.590.112
Glass bottles for EDTA	n.p.	n.p.
Clean/autoclaved glass beakers	histocom (Epredia)	3052835
Brushes, (for placement of section on capture area), fine	n.p.	n.p.
Brushes, (for disconnecting paraffin sections), middle	n.p.	n.p.
Brushes, (for water), coarse	n.p.	n.p.
Brushes, (for paraffin), coarse	n.p.	n.p.
Superfrost slides	Thermo Scientific	J1800AMNZ
Coverslips	VWR	631-0146
Microtome blade	Epredia	MX35 Premier disposable microtome blades, low profile
Microtome blade	Feather	S35 Microtome blade, stainless steel

n.p.: no preference.

## Step-by-step method details

### Major Step 1: Sample treatment and paraffin embedding (for workflow see Figure 1)

**Timing: 12 days**

**Figure 1:**
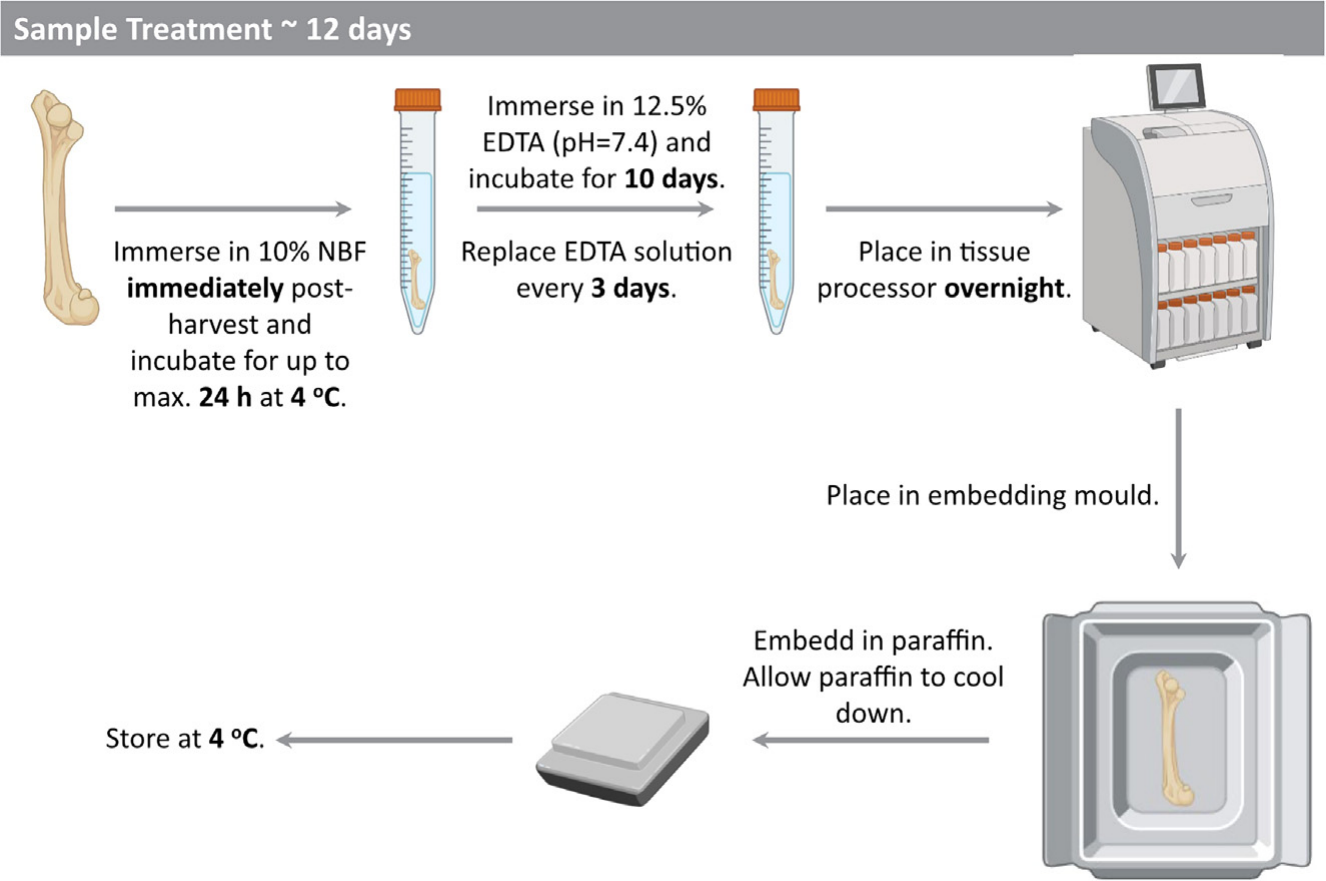
Overview of sample treatment and paraffin embedding workflow consisting of fixation, decalcification, paraffin infiltration and paraffin embedding Created with BioRender.com.

Major Step 1 describes the fixation, decalcification and embedding of bone and multi-tissue musculoskeletal samples from mice. Special considerations when processing samples from externally-stabilized femur defect models in mice are indicated in the notes related to sub-step 6.

1. Fix the tissue in 10% neutrally buffered formalin for up to 24 h (ca. 16–24 h) at 4°C.
2. Decalcify the tissue in pre-chilled 12.5% EDTA (pH 7.5) for 10 days at 4°C.
3. During decalcification exchange the EDTA every 3 days.
4. Transfer sample with tweezer into histology cassette and incubate in Ethanol (increasing Ethanol conc. 30%, 50%, 70% for 1 h each).

***Optional:*** If wanted bone and muscle can be carefully separated with scissors and placed into separate cassettes

5. Place histology cassettes into tissue processor with the following program: 1. Flushing with 70% Ethanol at 37°C for 1 h, 2. Flushing with 70% Ethanol at 37°C for 1 h, 3. Rinsing with 100% Ethanol at 37°C for 30 min, 4. Rinsing with 100% Ethanol at 37°C for 30 min, 5. Dehydration in 100% Ethanol at 37°C for 1 h, 6. Clearing with Isopropanol at 40°C for 1 h, 7. Clearing with Isopropanol at 45°C for 1 h, 8. Infiltration with Paraffin at 62°C for 4.5 h.
6. Remove samples from tissue processor and proceed to Paraffin embedding.

***Note:*** for muscle tissue: cross-sections are easier to cut compared to longitudinal sections.

***Note:*** for bone tissue: aligning the femur axis to the embedding mold allows for easier sectioning compared to oblique embedding.

***Note:*** if external fixation is used to study bone healing: for embedding of bones with the external fixator still being attached, hold the external fixator with tweezers while embedding. Fill Paraffin until the bone is covered without yet reaching the fixator body, do not let the Paraffin completely dry. Carefully remove the pins of the fixator, completely fill the mold with paraffin and place the histology cassette on top of the paraffin.

7. Place mold on 4°C plate for several hours (ca. 3–7 h).
8. Remove FFPE sample from mold and store at 4°C in fridge – Pause point (days-years).

### Major Step 2: FFPE sample selection and preparation for sectioning (for workflow see Figure 2A)

**Timing: 1 day**

**Figure 2:**
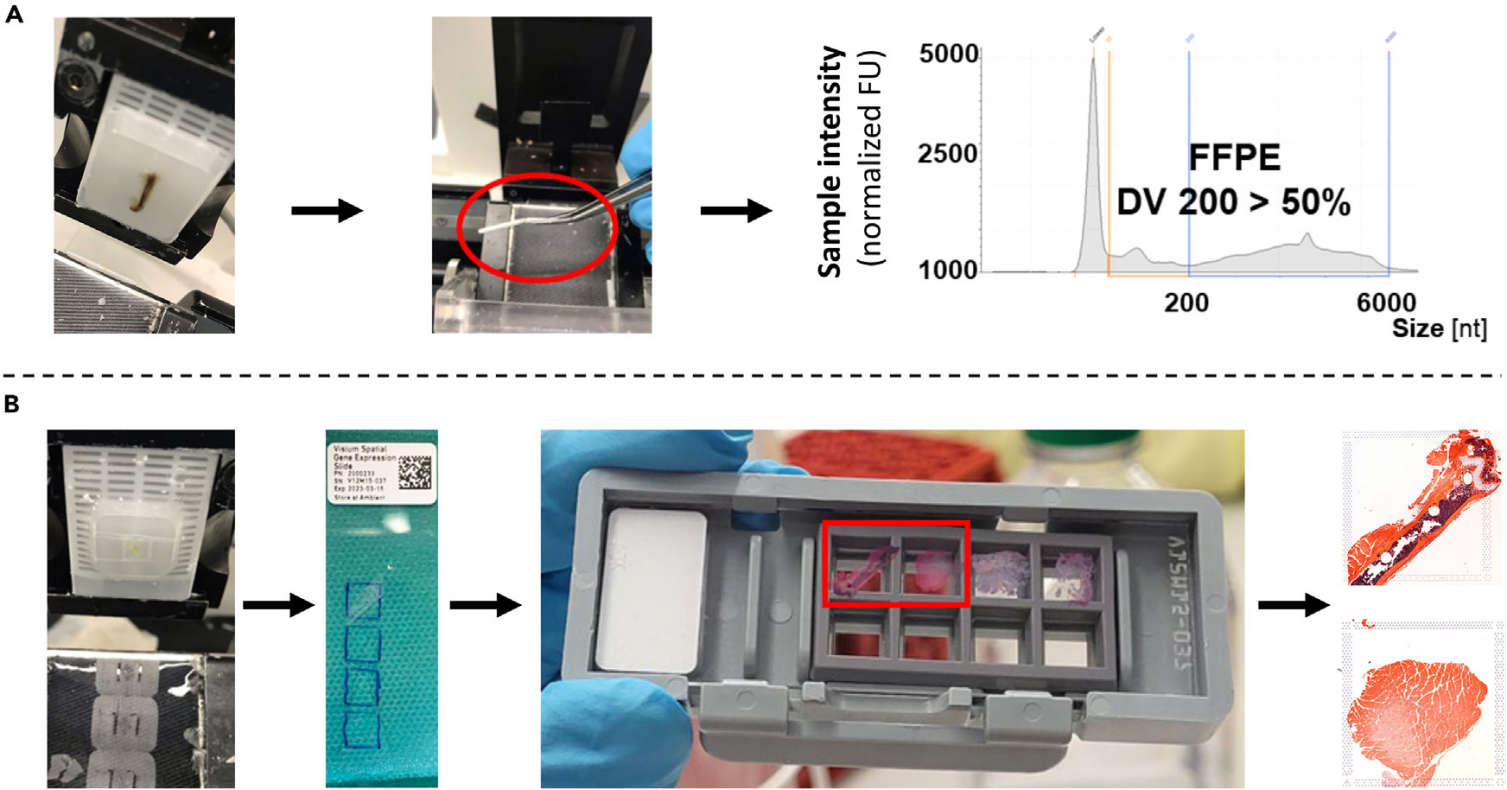
Workflow for spatial transcriptomics of musculoskeletal FFPE samples from mice (A) Pre-treatment: Sectioning, pooling and RNA quality check (B) Spatial transcriptomics: section scoring, placement on capture area, H&E staining and imaging prior to library prep and RNA-seq.

Major Step 2 describes the sample selection based on RNA quality as well as the preparation for sectioning of bone and multi-tissue musculoskeletal samples from mice. The steps are based on the Visium FFPE tissue preparation guide available at: https://www.10xgenomics.com/support/spatial-gene-expression-ffpe/documentation/steps/tissue-prep/visium-spatial-gene-expression-for-ffpe-tissue-preparation-guide.

9. Place FFPE block on 4°C plate or on metal block in freezer (<5 min).
10. Set-up microtome and insert blade.
11. Clamp FFPE block in microtome.
12. Align complete surface of FFPE block to the blade (90°).
13. Trim FFPE block until all tissues on block are exposed/representative of tissues later to be placed on the Visium slide.
14. Cut a section of 10 μm (without water flow turned on) and immediately place the rolled-up section with autoclaved tweezers into a pre-cooled autoclaved Eppendorf tube.
15. Repeat step 14 3x, to pool 4 sections of 10 μm per FFPE tissue block in one Eppendorf tube.
16. Immediately proceed to RNA isolation or intermediately store the Eppendorf tube with the pooled sections at −80°C – Pause point (days – weeks).
17. Isolate RNA using the RNeasy FFPE kit (QIAGEN) – Pause point.
18. Immediately proceed to DV200 measurements or intermediately store the RNA at −80°C – Pause point (days – weeks).
19. Determine the DV200 using a TapeStation and select samples meeting the requirements of the specific spatial transcriptomics platform (e.g., DV 200 ≥ 50% for Visium FFPE spatial transcriptomics) – Pause point (days – weeks).

### Major Step 3: Sample sectioning and placement on visium FFPE slide (for workflow see Figure 2B)

**Timing: 1 day**

Major Step 3 provides details on the sample sectioning and placement on the Visium FFPE slide. We have included detailed steps relevant for processing bone and multi-tissue musculoskeletal samples from mice.

20. Wipe microtome, water bath and all surfaces with RNase away.
21. Thoroughly flush with Milli-Q water.
22. Set-up microtome and water bath for Paraffin sections (42°C) using Milli-Q water.
23. Place a lamp next to water bath to allow bright illumination during the sectioning + sample placement on the Visium slide.
24. If the sample is bigger than the capture area carefully score sample using a scalpel blade (no. 11 or 15, stored at room temperature, ca. 20°C) or a microtome blade (stored at room temperature, ca. 20°C).

***Note:*** Try to have Paraffin within the scored area to allow for easier section handling in the water bath.

***Note:*** If possible, try to have one end of the bone (condyles, femoral head) intact, to avoid disintegration and detachment of the tissue during the Visium workflow.

25. Place selected and scored Paraffin blocks in an ice water bath (using Milli-Q water) as described in Visium protocol (sample surface facing down, ca. 5–10 min).
26. Clamp sample into microtome and carefully re-align surface of pre-cut FFPE block with microtome blade (MX35, Epredia).
27. Trim sample until cuts are intact (10 μm thickness).
28. Remove Paraffin debris with dedicated brush (do not use for other manipulations).
29. Connect water slide to water bath and place section with brush on Superfrost slide.
30. Check section for integrity under microscope.
31. Cut an additional section at 10 μm thickness, directly followed by a section at 5 μm (to be used on Visium slide).
32. Let the sections slide into the water bath and immediately disconnect the waterflow to allow easier handling of the sections in the water bath.
33. Remove any attached Paraffin and tissue outside the scoring area with two fine brushes.
34. Let the section float until wrinkles disappear.
35. Visually check section on integrity, particularly at the interface of different tissues (e.g., bone and muscle).
36. Mark capture areas on back of Visium slide with water resistant marker.
37. Place the 5 μm section with a fine brush on the capture area with all tissue within the capture area while Paraffin can extend outside the capture area.
38. Repeat for three additional sections/capture areas.

### Major Step 4: Drying, staining, imaging and decrosslinking (for workflow see Figure 2B and manual from 10× genomics online, H&E images shown in Figures S1 and S2)

**Timing: 1 day**

39. Perform all steps according to the Visium protocol CG000409| Rev D: Demonstrated Protocol | Visium Spatial for FFPE – Deparaffinization, H&E Staining, Imaging & Decrosslinking CG000409 | Rev D.

***Note:*** Use sterile tweezers when handling the Visium slides to avoid impact on RNA quality.

**CRITICAL:** For the staining of musculoskeletal tissue sections from mice, take care to slowly transfer the slide from one solution to the next to avoid detachment of the tissue.

### Major Step 5: Probe hybridization, ligation, library prep and sequencing (for workflow see manual from 10× genomics online)

**Timing: < 7 days**

40. Perform all steps according to the Visium protocol CG000407| Rev E including the QCs: User Guide | Visium Spatial Gene Expression for FFPE CG000407 | Rev E.

**CRITICAL:** For the post hybridization and ligation washes take care to slowly add the wash buffer to the side of the gasket to avoid detachment of the tissue.

### Major Step 6: 10x Visium spatial transcriptomic data analyses and visualization (for workflow see Figure 3)

**Timing: 1 day**

41. Install software and dependency packages, download the reference genome, probe set reference file, and the slide layout GPR file for the mouse model from the weblink provided under software and dependency requirements.
42. Use Space Ranger’s mkfastq module to perform the process of separating Visium-prepared raw base call (BCL) files produced by Illumina sequencers into FASTQ files.
43. Align the unprocessed Visium sequence data to the mouse reference genome, then generate spatial features, count matrices, and filter barcodes using the count pipeline within Space Ranger.
44. Perform spot-level expression data analysis using Seurat (R toolkit for single-cell genomics)^11^ or Scanpy (Python-based platform).^12^
45. Perform quality control and basic filtering of spots based on total counts and expressed genes. Further exclude spots with mitochondrial and ribosomal reads from the analysis.
46. Normalize and scale the data and identify significant variable genes using SC Transform.^13^
47. Apply the dimensionality reduction technique to the dataset and elbow plots to identify the appropriate number of principal components for clustering.
48. To identify clusters of spots, employ a clustering algorithm that optimizes shared nearest neighbor (SNN) modularity.
49. Visualize spots clustering using uniform manifold approximation and projection for dimension reduction (umap).^14^
50. Overlay umap gene expression clusters with histology image.
51. Perform further downstream analysis such as clustering and visualization, cell-specific marker genes, cell-type identification, segmentation and visualization in the spatial domain, spatial trajectory inference, cell-cell interaction, identification of spatially variable genes, differential gene expression analysis, enrichment analysis of spatial expression data etc.
52. Perform tissue annotation using qupath (https://qupath.github.io/https://qupath.github.io/) and extract the spot coordinates and barcodes using Loupe Browser (https://www.10xgenomics.com/support/software/loupe-browser/latest).

**Figure 3:**
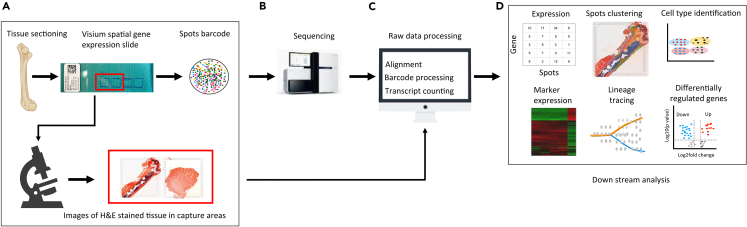
Comprehensive analysis pipeline for spatially-resolved transcriptomics data (A) The tissue sections are placed on the Visium slide. Barcoded capture probes store spatial information, which is added to the captured transcript before sequencing. Stained tissue sections are examined by microscopy to acquire bright field imaging. (B) Sequence whole-transcriptome gene expression libraries on sequencing instrument. (C) The sequencing data is used as input for demultiplexing and transcript quantification using space ranger pipeline. (D) The count data, together with the image data, are used as inputs to perform various downstream analyses.

## Expected outcomes

The developed protocol enables the application of FFPE musculoskeletal tissue samples from mice to spatial transcriptomics. Our motivation was to assess spatially-resolved gene expression within fractured bone and the surrounding muscular tissue in FFPE samples from mice. To achieve this, we developed a spatial transcriptomics protocol for musculoskeletal tissue overcoming current restrictions in mineralized samples. The protocol allows to meet quality measures (e.g., DV 200 can be kept >50 for bone+/- muscle during decalcification), we highlight critical steps (timing of tissue harvest; tissue embedding and sample orientation important for later sectioning) and indicate instruments and tools helpful for the processing and the application of musculoskeletal tissue to spatial transcriptomics. Samples processed according to this protocol can be integrated into further workflows, e.g., multimodal approaches for comprehensive locally and spatially-resolved understanding of tissue (patho)physiology.^15^^,^^16^^,^^17^

## Limitations

The protocol has been established for musculoskeletal samples from mice. All pretreatment steps, including the decalcification period, were optimized for samples from an externally-stabilized femur defect model in mice. While we have also used this protocol for bone samples (vertebra, intact femur), muscle samples (m. quadriceps femoris) and multi-tissue samples (intact femur with adjacent muscle) from mice, application of the protocol to other musculoskeletal sites and species may require further optimization (e.g., addition of RNase inhibitor during sample pretreatment).

## Troubleshooting

### Problem 1

Inhomogeneity in Paraffin of the embedded samples (related to Step 1). This can occur when the Paraffin was added several times with the first layers already being cooled down when further layers were added.

### Potential solution

• Make sure to have no phase separation of the Paraffin during embedding.
• Do not let layers of Paraffin cool down before adding further Paraffin

### Problem 2

Low DV 200 ratio (related to Step 2). This can be caused by the pre-treatment, the duration of sample storage and the exposure to RNases during sectioning.

### Potential solution

• Shorten the pre-treatment by determining the minimum required decalcification time to allow sectioning of the samples.
• Avoid long-term storage.
• Use sterile instruments and RNase away.
• Switch to spatial transcriptomics workflows targeted towards transcriptome recovery from degraded samples.

### Problem 3

Disintegration of tissue during cutting and floating (related to Step 3). This can be caused by the scoring lines not being optimal and drying out of the tissue block.

### Potential solution

• Use a new scalpel blade or a microtome blade for scoring.
• Try to have one side of tissue intact/not scored.
• Extension of the Paraffin on one side allows for easier handling.
• Re-incubate the Paraffin block in the ice water bath.
• Further solutions can be found in CG000408.

### Problem 4

Detachment of the tissue section from the spatial slide (related to Steps 4 and 5). This can be caused by having several tissues on one section with intersections being prone to detachment, short drying periods and the scoring lines not being optimal.

### Potential solution

• Make sure the sections are sufficiently dried (3 h at 42°C plus overnight drying in dessicator/with dessicants in Falcon tube at room temperature).
• Use a new scalpel blade or a microtome blade for scoring.
• Try to have one side of tissue intact/not scored.
• Extension of the Paraffin on one side allows for easier handling.
• Further solutions can be found in CG000408.

### Problem 5

Voids in between tissues on the histology sections (related to Steps 4 and 5). This can be caused by using only partially decalcified samples and drying of the sections during the staining process.

### Potential solution

• Make sure to use decalcified samples, e.g., by checking decalcification of similarly treated samples by micro-computed tomography.
• Use new microtome blade.
• Strictly keep staining and drying times.

## Resource availability

### Lead contact

Further information and requests for resources and reagents should be directed to and will be fulfilled by the lead contact, Esther Wehrle (esther.wehrle@aofoundation.org).

### Technical contact

Technical questions about this protocol should be directed to the technical contact, Esther Wehrle (esther.wehrle@aofoundation.org).

### Materials availability

There are no newly generated materials associated with this protocol.

### Data and code availability

The data are available from the corresponding author on request.
