# Supplementary material for: Protocol for preparing formalin-fixed paraffin-embedded musculoskeletal tissue samples from mice for spatial transcriptomics
Source: STAR Protoc. 2024 Mar 29;5(2):102986. doi: 10.1016/j.xpro.2024.102986 (PMC10998190; doi:10.1016/j.xpro.2024.102986)
Supplement: Document S1. Figures S1 and S2 [file mmc1.pdf]

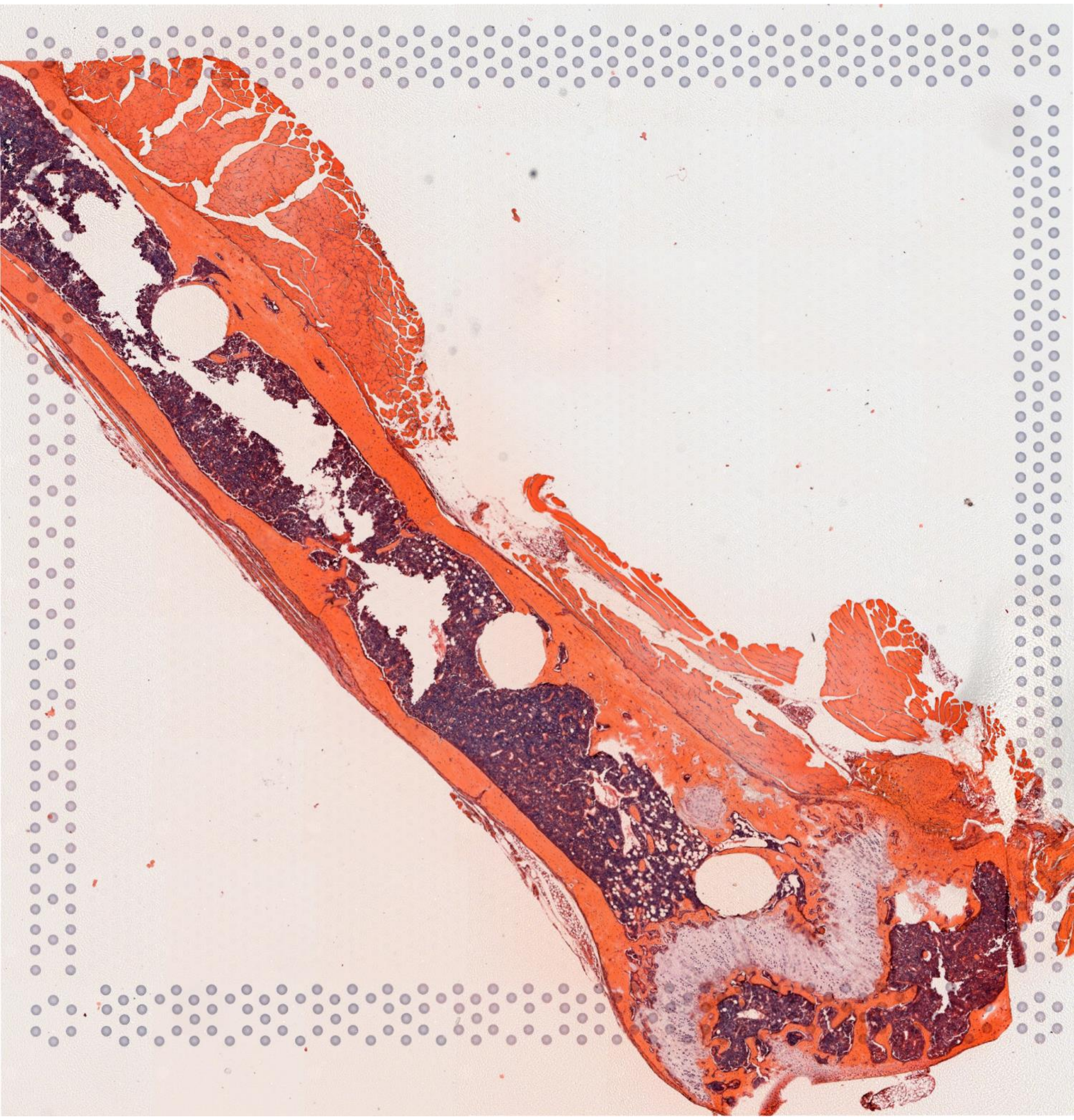

**Supplementary Figure 1.** H&E stained mouse femur with adjacent muscle within the 6.5x6.5mm capture area on a Visium FFPE spatial transcriptomics slide, related to Step 4.
Images taken with Zeiss Polarization Microscope Axio Imager Z.2.

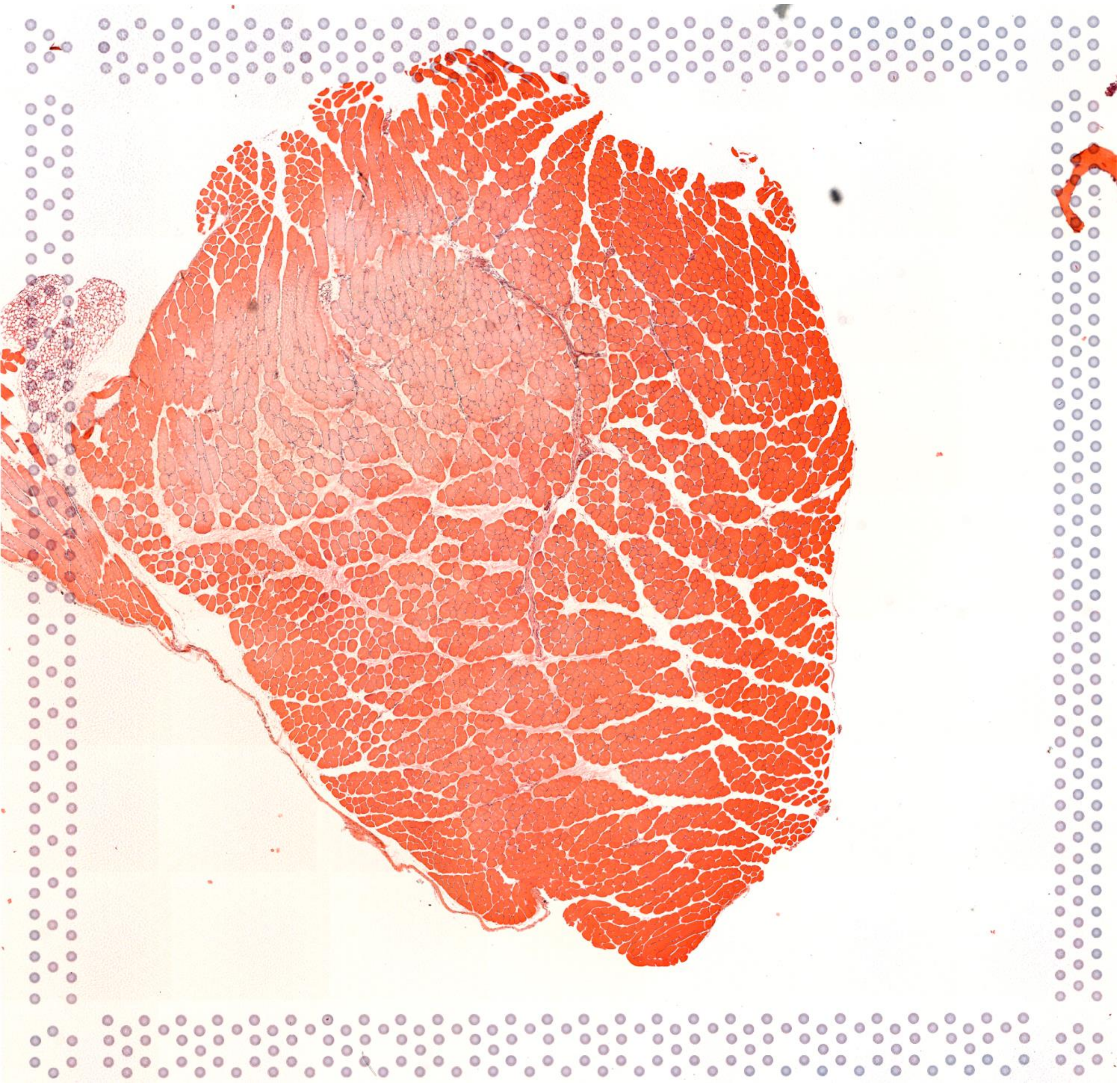

**Supplementary Figure 2.** H&E stained mouse m. quadriceps femoris within the 6.5x6.5mm capture area on a Visium FFPE spatial transcriptomics slide, related to Step 4.
Images taken with Zeiss Polarization Microscope Axio Imager Z.2.
